# Anhydroicaritin Inhibits EMT in Breast Cancer by Enhancing GPX1 Expression: A Research Based on Sequencing Technologies and Bioinformatics Analysis

**DOI:** 10.3389/fcell.2021.764481

**Published:** 2022-02-01

**Authors:** Feifei Li, Youyang Shi, Xiaojuan Yang, Zhanyang Luo, Guangtao Zhang, Kui Yu, Feng Li, Lixin Chen, Youkang Zhao, Ying Xie, Yuanyuan Wu, Jianfeng Yang, Xiqiu Zhou, Sheng Liu

**Affiliations:** ^1^ Longhua Hospital, Shanghai University of Traditional Chinese Medicine, Shanghai, China; ^2^ Department of Surgery, Pudong Branch of Longhua Hospital, Shanghai University of Traditional Chinese Medicine, Shanghai, China; ^3^ Department of Gastroenterology, Naval Medical Center of PLA, Naval Military Medical University, Shanghai, China

**Keywords:** RNA sequencing, bioinformatics analysis, anhydroicaritin, breast cancer, GPx1, epithelial to mesenchymal transformation

## Abstract

**Background:** Breast cancer (BC) is the leading cause of cancer-related deaths among women worldwide. The application of advanced technology has promoted accurate diagnosis and treatment of cancer. Anhydroicaritin (AHI) is a flavonoid with therapeutic potential in BC treatment. The current study aimed to determine AHI’s mechanism in BC treatment *via* RNA sequencing, comprehensive bioinformatics analysis, and experimental verification.

**Methods:** Network pharmacology and MTT (3-(4,5)-dimethylthiazolyl-3,5- diphenyltetrazolium bromide) experiments were conducted to first confirm AHI’s anti-BC effect. RNA sequencing was performed to identify the genes affected by AHI. Differential expression analysis, survival analysis, gene set enrichment analysis, and immune infiltration analysis were performed *via* bioinformatics analysis. Western blot analysis, reverse transcription–polymerase chain reaction (RT-PCR) experiment, molecular docking, and drug affinity responsive target stability (DARTS) experiments were also performed to confirm AHI’s direct effect on glutathione peroxidase 1 (GPX1) expression. Confocal immunofluorescence analysis was conducted to verify AHI’s effect on the occurrence and development of epithelial–mesenchymal transition (EMT). Finally, BC nude mouse xenografts were established, and AHI’s molecular mechanism on BC was explored.

**Results:** Network pharmacology results demonstrated that AHI’s therapeutic targets on BC were related to the proliferation, invasion, and metastasis of BC cells. AHI significantly inhibited the proliferation of 4T1 and MDA-MB-231 BC cells in the MTT experiments. RNA sequencing results showed that AHI upregulated the GPX1 expression in the 4T1 and MDA-MB-231 BC cells. Next, bioinformatics analysis revealed that GPX1 is less expressed in BC than in normal breast tissues. Patients with high GPX1 expression levels tended to have prolonged overall survival and disease-free survival than patients with low GPX1 expression levels in BC. Western blot and RT-PCR experiments revealed that AHI increased the protein and mRNA levels of GPX1. Molecular docking and DARTS experiments confirmed the direct binding combination between AHI and GPX1. After the evaluation of the EMT scores of 1,078 patients with BC, we found a potential anti-BC role of GPX1 possibly *via* suppression of the malignant EMT. The confocal immunofluorescence analysis showed that AHI increased E-cadherin expression levels and reduced vimentin expression levels in BC cells. Animal experiments showed that AHI significantly inhibited tumor growth*.* AHI also inhibited EMT by enhancing GPX1 and caspase3 cleavage, hence inhibiting EMT markers (i.e., N-cadherin and vimentin) and Ki-67.

**Conclusion:** GPX1 plays a critical role in BC, which may be a biomarker for the prognosis. In addition, AHI suppressed EMT by increasing GPX1 expression, which may serve as a potential therapy for BC treatment.

## Introduction

The global cancer statistics report predicted that new cases of breast cancer (BC) would reach 2.3 million in 2020, accounting for 11.7% of all new cancers. BC has become the most common cancer overall (Sung et al., 2021). The treatment of BC has developed into a multidisciplinary comprehensive diagnosis and treatment model, which includes surgery, chemotherapy, radiotherapy, endocrine therapy, targeted therapy, and immunotherapy.

The advantages of traditional Chinese medicine (TCM) have gradually emerged with its core ideas of holistic view and syndrome differentiation-based treatment. TCM has played a critical role in BC prevention and treatment. The previous studies of our team have confirmed that TCM has apparent advantages in improving the quality of life of patients, reducing the toxicity of chemotherapy, and prolonging disease-free survival (DFS) (Hao et al., 2020; Wang Y. et al., 2020). Anhydroicaritin (AHI) is a flavonoid with antitumor biological and pharmacological effects (Nguyen et al., 2017). AHI is the primary metabolic product of *Epimedium brevicornum* after digestion and absorption (Zhou et al., 2021). Glutathione peroxidase 1 (GPX1) is a vital antioxidant enzyme that can catabolize the hydrogen oxide entering the human body and reduce peroxide’s damage to the body (Huang et al., 2018). As one of the important cell biology processes, epithelial–mesenchymal transition (EMT) plays a key role in multiple links of tumor metastasis (Wilson et al., 2021; Yang et al., 2021). A previous study also revealed that GPX1 inhibits EMT progression in cancer and plays an anticancer role (Meng et al., 2018). However, research on the application of AHI and GPX1 in BC treatment remains limited, especially at the transcriptome level and the molecular mechanism.

**FIGURE 1:**
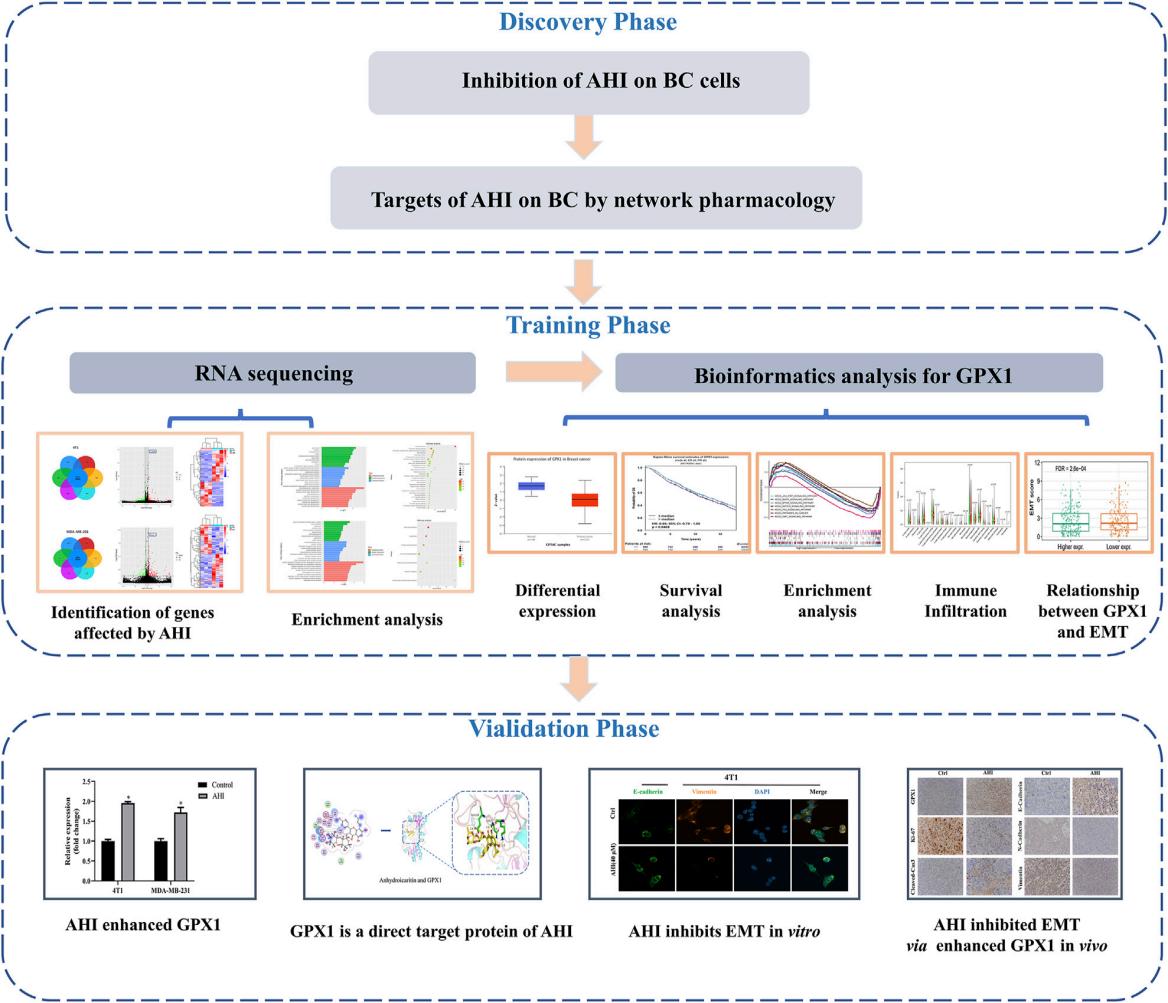
Workflow chart of the research.

The present study is an in-depth research on the anti-BC mechanism of AHI *via* RNA sequencing, bioinformatics analysis, network pharmacology, molecular docking, and *in vitro* and *in vivo* experiments. Network pharmacology and MTT (3-(4,5)-dimethylthiazolyl-3,5- diphenyltetrazolium bromide) experiments demonstrated the anti-BC potential of AHI. RNA sequencing revealed that AHI enhanced GPX1 in 4T1 and MDA-MB-231 BC cells. Comprehensive bioinformatics analysis showed that GPX1 is closely related to the prognosis of BC patients. Western blot analysis, reverse transcription–polymerase chain reaction (RT-PCR), molecular docking, and drug affinity responsive target stability (DARTS) experiments confirmed that AHI directly affects GPX1 expression. Animal experiments showed the anti-BC effect of AHI, possibly through the enhancement of GPX1 expression and thus EMT inhibition. The detailed strategy of this study is shown in Figure 1.

## Materials and Methods

### Network Pharmacology Analysis

The targets of AHI were searched in the TCMSP (http://tcmspw.com/tcmsp.php) (Ru et al., 2014), Swiss target prediction (http://www.swisstargetprediction.ch/) (Daina et al., 2019), PubChem, TargetMol, and BioCrick databases. The names of the target proteins were uniformly converted into gene names using UniProt database (http://www.uniprot.org/) (Rovatsos et al., 2019). BC-related targets were collected and screened *via* GeneCards database (https://www.genecards.org/). DAVID database (https://david.ncifcrf.gov/home.jsp) was used to analyze Kyoto Encyclopedia of Genes and Genomes (KEGG) pathway enrichment (Jiao et al., 2012). The AHI–targets–pathways network was constructed using Cytoscape 3.7.2.

### Cell Cultures

Two BC cell lines, including murine 4T1 cells (TNBC, highly metastatic) and MDA-MB-231 (TNBC), were obtained from Chinese Academy of Sciences (Shanghai, China). 4T1 cells were maintained in RPMI 1640 medium supplemented with 10% (vol/vol) fetal bovine serum (FBS), 100 U/mL penicillin, and 100 mg/L streptomycin. MDA-MB-231 cells were maintained in Dulbecco modified eagle medium (DMEM) supplemented with 10% FBS, 100 U/mL penicillin, and 100 mg/L streptomycin. The cells were incubated at 37°C in a 5% CO_2_ incubator with saturated humidity.

### Cell Proliferation Assays

**FIGURE 2:**
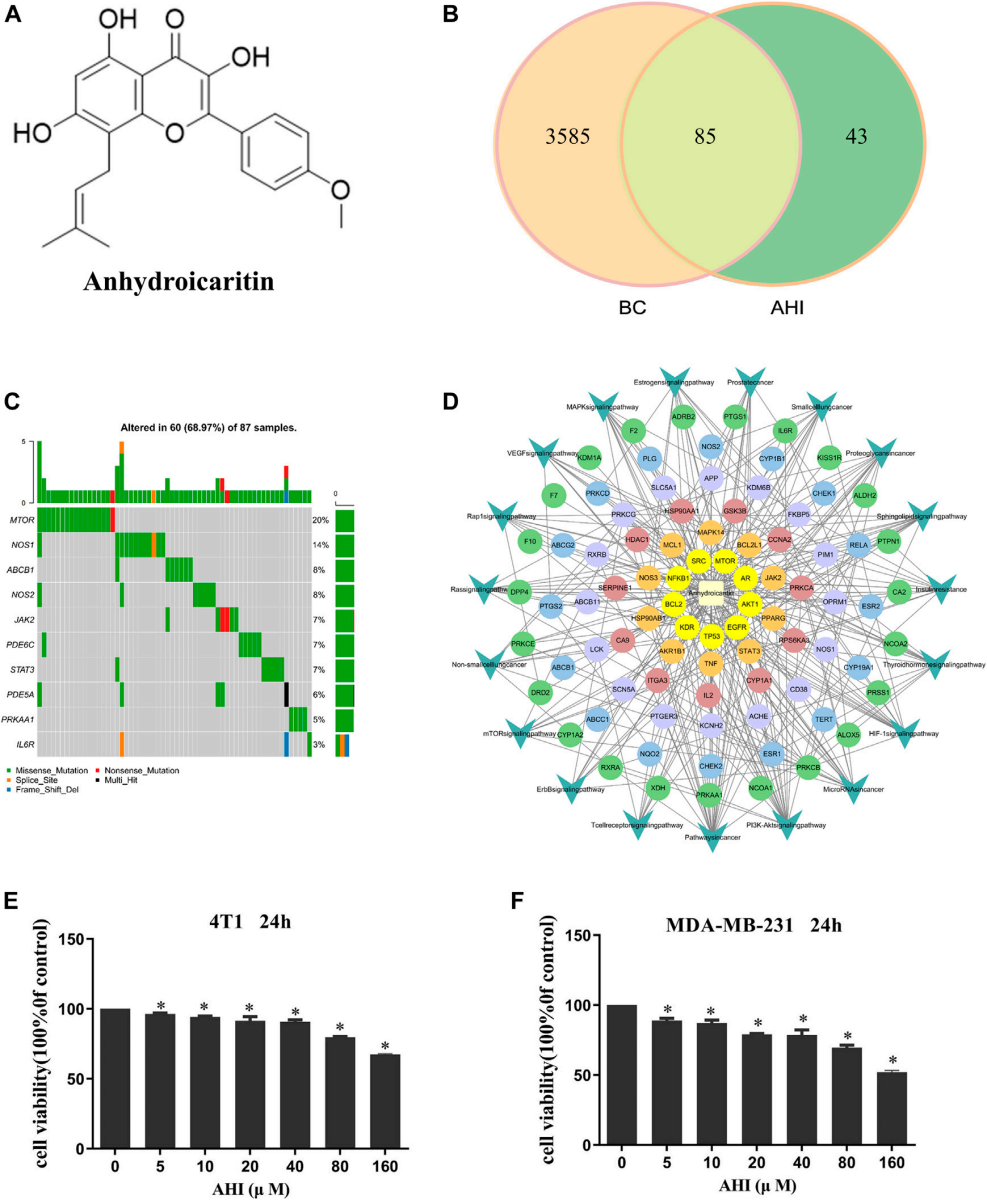
AHI treatment potential in BC. **(A)** Chemical structure of AHI. **(B)** Venn diagram depicting the number of potential targets of AHI for BC treatment. **(C)** Waterfall map of the mutation information (TCGA-downloaded BC data). **(D)** AHI–targets–pathways network. (The circle represents the targets, and the arrow represents the pathways.) **(E**, **F)** AHI inhibited the survival of the 4T1 and MDA-MB-231 cells at different concentrations (24 h). *p < 0.05.

AHI (Figure 2A) was purchased from Shanghai Weiao Medical Technology Co., Ltd. (Shanghai, China). 4T1 and MAD-MB-231 cells (5 × 10^3^/well) were separately seeded into 96-well plates. After 24 h, the culture medium was replaced with fresh medium containing various concentrations of AHI (0, 5, 10, 20, 40, 80, and 160 µM). After incubation for 24 h, cell viability was detected by MTT assay. Values of OD490 nm were measured using a BioTek instrument (Winooski, VT, USA). Data are expressed as the mean ± SD of at least three independent experiments.

### RNA Extraction and Sequencing

After treatment of the 4T1 and MDA-MB-231 cells with AHI (40 μM), RNA from the 4T1 and MDA-MB-231 cells treated with the optimal concentration of AHI was extracted using Trizol reagent. To ensure that the samples were qualified and appropriate for sequencing, Nanodrop, Qubit 2.0, and Agilent 2100 were used to detect the purity, concentration, and integrity of the RNA samples, respectively. The sample’s optical density 260/280 must be greater than 1.8, and the sample has to be free of protein or visible impurities. Library construction and RNA sequences were performed according to the manufacturer’s instructions. RNA sequencing was performed on the 4T1 and MDA-MB-231 cells using NovaSeq. Construction of the sequencing library and RNA sequencing were performed by Sangon (Shanghai, China) using Illumina No-voseq Platform. Differentially expressed genes (DEGs) were calculated *via* DESeq version 1.30.0. The *p* value was adjusted according to the false discovery rate (FDR). *p* < 0.05 and fold change ≥1.5 were set to be statistically significant. Gene Ontology (GO) functional enrichment and KEGG pathway analyses were performed for the bioinformatics analysis.

### Analysis of Gene Expression Differences and Prognostic Value

The protein expression of GPX1 in BC was evaluated through CPTAC analysis (http://ualcan.path.uab.edu/) (Chen et al., 2019). The prognostic value of GPX1 was assessed using Breast Cancer Gene-Expression Miner version 4.7r (http://bcgenex.centregauducheau.fr/BC-GEM) (Jézéquel et al., 2012).

### Protein Interaction Relationship and Gene Set Enrichment Analysis

The upstream and downstream protein regulatory relationship of GPX1 was predicted by STRING database (https://string-db.org/cgi/input.pl) (Szklarczyk et al., 2019). The enrichment pathways of the gene in each phenotype were ranked using the gene set enrichment analysis (GSEA) calculation method (Subramanian et al., 2007). The FDR value and normalized enrichment score (NES) were used to evaluate whether the pathway enrichment is significant (FDR <0.05 and NES >0.25).

### Immune Infiltration Analysis

The gene expression profiles of 1,053 BC tissues and 111 normal breast tissues were downloaded from The Cancer Genome Atlas (TCGA) database (Blum et al., 2018). CiberSort algorithm was used to evaluate the correlation between the gene expression and the immune infiltration of 22 immune cell subtypes. The relationships among gene and B cells, CD4^+^ T cells, CD8^+^ T cells, neutrophils, macrophages, and dendritic cells in BC were evaluated through the deconvolution method of TIMER database (Li et al., 2017). The gene module used the relationship between the gene expression and the abundance of immune infiltration.

### RT-PCR

Total RNA was isolated using Trizol reagent. The first strand of cDNA was synthetized using a reverse transcription kit (PrimeScript™ Synthesis Kit; Takara Bio, Inc., Dalian, China). RT-PCR experiment was performed using SYBR Premix Ex Taq Kit (Takara Bio, Inc.) on an Applied Biosystems 7500 Real-Time PCR System (Applied Biosystems, White Plains, NY, USA). The GAPDH was used as internal control. Primers for GPX1 and GAPDH are presented in Supplementary Table S1.

### Western Blot Analysis

After the 4T1 and MDA-MB-231 cells were treated with AHI (0, 10, 20, or 40 µM) for 24 h, total cell protein lysates were extracted using RIPA lysis buffer that contained protease and phosphatase inhibitor cocktails. As determined *via* BCA analysis (Beyotime Biotechnology, Shanghai, China), protein lysates (20 µg) were then subjected to Western blot analysis. The primary antibody used in the analyses was GPX1 (1:1,000; ABclonal Technology, Wuhan, China). The secondary antibody was a goat anti-rabbit secondary antibody coupled to horseradish peroxidase (1:5,000; Proteintech, China). The target protein bands were visualized using a chemiluminescent kit (Beyotime Biotechnology) and analyzed using Image Lab software.

### Molecular Docking

The energy of the compound downloaded from the PubChem database was minimized through Chem3D. We then converted the downloaded compound to the mol2 format. The target’s crystal structure was downloaded from PDB database (https://www.rcsb.org/) (Burley et al., 2021). The additional process was undertaken using Pymol 2.1 and AutoDockTools 1.5.6. The treated compound was used as a small molecule ligand, and the target was used as a receptor. Molecular docking was conducted using AutoDockTools.

### DARTS Analysis

Cells were exposed to AHI (40 μM) and DMEM for 3 h and then lysed using an NP-40 lysis buffer containing phosphatase and complete protease inhibitors. The protein concentration was measured using a BCA protein assay reagent. Pronase was then added with the ratios of 1:2,000 and 1:3,000 (pronase/total protein), and the mixtures were then incubated at room temperature for 30 min. Subsequently, the reaction products were analyzed *via* Western blot assays.

### Analysis of the Relationship Between GPX1 and EMT

Tumor RNA-seq data for the 1,078 patients with BC were downloaded and normalized from the TCGA and gene expression omnibus databases. Student *t* test was performed to compare the statistical difference between the high and low GPX1 expression groups using ggplot2 R package. The EMT scores for individual cases were assessed as the average expression of genes upregulated in EMT minus the average expression of genes downregulated in EMT using 17 EMT signatures (i.e., ZEB1, ZEB2, Snai1, Snail2, Twist1, Twist2, FOXC2, VIM, FN1, SOX10, MMP2, MMP3, CDH1, CLDN3, CLDN4, CLDN7, and DSP) [PMID: 26245467]. Spearman correlation analysis was performed to evaluate the correlation between GPX1 expression and the EMT signatures without a normal distribution using ggstatsplot R package. The MCPCOUNTER algorithm was used to score 1,097 BC samples for immune cell infiltration. Statistical analyses were performed using R software version 4.0.3 (R Foundation for Statistical Computing, Vienna, Austria). *p* < 0.05 was considered statistically significant.

### Confocal Immunofluorescence Assay

When the 4T1 and MDA-MB-231 cells seeded in the confocal plates grew into 60% confluence, they were washed with phosphate-buffered saline (PBS), fixed in 4% paraformaldehyde for 5 min, permeabilized with 0.5% Triton X-100 for 5 min, and blocked with bovine serum albumin for 1 h at room temperature. The cells were then incubated with primary antibodies at 4°C overnight, followed by incubation with fluorophore-conjugated secondary antibody for 1 h. After washing, the samples were stained with DAPI and imaged using a confocal microscope. The primary antibodies were E-cadherin (1:100; Cell Signaling Technology 14472) and vimentin (1:100, A19607; ABclonal Technology).

### Establishment of Animal Xenografts

All animal experiments were performed according to protocols approved by the Animal Care and Use Committee of Shanghai University of TCM. Six-week-old female nude mice (SLAC Laboratory Animal Co., Ltd., Shanghai, China) were anesthetized, and the human TNBC cell line MDA-MB-231 (1 × 10^7^) premixed with Matrigel at a ratio of 1:1 was subcutaneously injected into the fourth pair of breast fat pads on the left side of each mouse. When the tumor approximately grew to 5 × 5 mm, the mice were randomized into the control group and AHI group (20 mg/kg) (n = 5). Mice received AHI treatment by intraperitoneal injection once every 2 days for 4 weeks. Tumor volumes were calculated using formula *V* = 0.5 × *a* × *b* ^2^, where *V* denotes tumor volume, *a* denotes maximum tumor diameter, and *b* denotes minimum tumor diameter. To assess the animals’ overall health, their body weight was measured once a week as an indicator. The mice were euthanized at the experiments’ termination. Tumors were isolated, imaged, weighed, and fixed with paraformaldehyde for further immunohistochemistry (IHC) evaluation.

### Hematoxylin–Eosin Staining

To better evaluate AHI’s anticancer effects, hematoxylin–eosin (H&E) staining was performed using a commercial H&E staining kit (Beyotime Biotechnology) following the manufacturer’s instruction. Paraffin-embedded tumor sections with thickness of 4 μm were fixed onto poly-l-lysine–coated slides dewaxed twice with xylene for 10 min each, followed by gradual rehydration in 100%–70% ethanol, and then immersion in distilled water. Subsequently, 10% hematoxylin was applied to stain nuclear for 5 min followed by counterstaining cytoplasm with eosin for 2 min. After dehydration, hyalinization, and sealing with neutral gum, the sections were baked, and pictures were taken and analyzed.

### IHC Analysis

For IHC analysis, the tumor tissues’ slides were deparaffinized twice with xylene for 10 min and rehydrated with 100%–75% ethanol for 10 min. After washing with PBS for three times, the slices were boiled in 10-mm sodium citrate buffer solution (pH 6.0; Solarbio, Beijing, China) for 8 min to perform antigen repair. To eliminate endogenous peroxidase activity, sections were permeabilized with 3% hydrogen peroxide dissolved in methanol at room temperature in the dark and then blocked by 10% goat serum to reduce nonspecific binding. The samples were then washed with PBS three times and incubated with a 1:100 diluted primary antibodies including Ki67 (CST, USA), cleaved caspase3 (CST, USA), GPX1 (ABclonal Technology, China), E-cadherin (CST, USA), N-cadherin (CST, USA), and vimentin (CST, USA) in a humid chamber at 4°C overnight, followed by incubation with a 1:200 dilution of biotinylated secondary antibodies. Immediately thereafter, 3,3-diaminobenzidine substrate (DAB, ZSGB-BIO, Beijing, China) was applied for color development, and a counterstain with Mayer’s hematoxylin was performed. Digital images of stained sections were taken using a BX46 Olympus microscope (Olympus, Center Valley, PA, USA).

## Results

### AHI’s Therapeutic Potential in BC Treatment

A total of 128 AHI-related targets were screened through the TCMSP database, Swiss target prediction, PubChem, TargetMol, and BioCrick databases. A total of 3,670 BC-related targets were screened through the GeneCards database. Taking the intersection, we obtained 85 potential targets of AHI intervention in BC treatment (Figure 2B). The maftools package of R language was used to count the mutation data downloaded from TCGA. The waterfall map shows that the mutation rates of target genes were generally low (Figure 2C). The network of AHI-targeted genes and signal pathways was constructed through Cytoscape (Figure 2D). The MTT experiment showed that the survival rates of the 4T1 and MDA-MB-231 cells gradually decreased with the increased concentration of AHI (Figures 2E,F). The IC_50_ values of AHI in the MDA-MB-231 cells were 278.68 μM at 24 h. The IC_50_ values for the 4T1 cells were 319.83 μM at 24 h. To avoid AHI’s cytotoxicity, the concentration of 40 μM, which is less than IC_50_, was used in the succeeding experiments.

### Transcriptome Profile of AHI-Treated BC Cells by RNA-Seq

**FIGURE 3:**
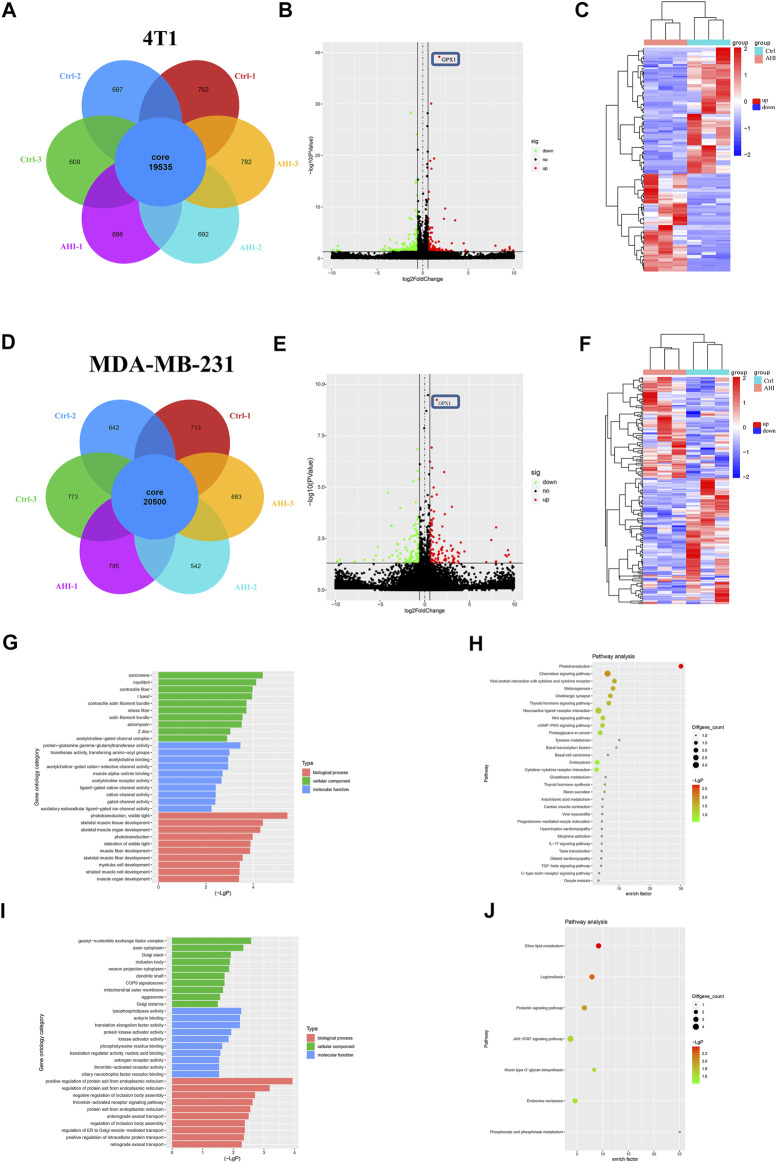
Screening of DEGs by RNA-seq and enrichment analysis. **(A**, **D)** Analysis of DEGs after AHI treatment. **(B**, **E)** Identification of DEGs. **(C**, **F)** Volcano plot of the DEGs with FDR ≤0.05 and fold change ≥1.5. (The red dots indicate upregulated DEGs, green ones indicate downregulated DEGs, and black ones indicate no significant difference. The heatmap was drawn to show the DEGs.) **(G**, **I)** GO functional enrichment and **(H**, **J)** KEGG pathway enrichment of the DEGs.

To further identify genes affected by AHI in BC treatment, the RNA of the 4T1 and MDA-MB-231 cells treated with or without 40 μM AHI were extracted for RNA sequencing. A total of 19,535 genes were identified as core genes and used in the analysis of the DEGs in the 4T1 cells (Figure 3A). A total of 135 DEGs were subsequently identified between the AHI-treated group and the control group according to the criteria of fold change ≥1.5 and *p* < 0.05. Compared with the control group, 59 genes were significantly upregulated, whereas 76 genes were significantly downregulated (Figure 3B). For the MDA-MB-231 cells, 20,500 genes were identified as core genes (Figure 3D). A total of 250 DEGs were subsequently identified between the AHI-treated group and the control group (Figure 3E). Compared with the control group, 144 genes were significantly upregulated, whereas 106 genes were significantly downregulated. AHI-treated 4T1 and MDA-MB-231 cells possessed genes with hierarchical clustering that are distinct from those in the control cells (Figures 3C,F). Regarding potential genes involved in AHI’s antitumor property, we found that GPX1 was the overlapping DEG in the 4T1 and MDA-MB-231 cells.

### Identification of Potential Biomarkers of AHI in Treating TNBC

To further explore the biological process and KEGG pathway affected by AHI in BC treatment, functional enrichment analysis was conducted for genes affected by AHI in BC treatment. GO functional enrichment was performed to explore the potential molecular or biological functions affected by AHI treatment. All the DEGs were categorized into the following three major categories: cellular components, molecular functions, and biological process. The DEGs in the AHI-treated group *versus* the control group in the 4T1 cells are significantly involved in sarcomere, myofibril, and contractile fiber, which are associated with tumor invasion and metastasis (Figure 3G), and their related signaling pathways, such as the Wnt signaling pathways and basal cell carcinoma (Figure 3H). Similarly, the GO biological processes significantly enriched by the AHI treatment in the MDA-MB-231 cells included axon cytoplasm, dendritic shaft (Figure 3I), and their related signaling pathways, such as JAK-STAT signaling pathways and mucin type O–glycan biosynthesis (Figure 3J). Considering the fundamental role of metastasis in BC pathogenesis, the current study inferred that GPX1 and signaling pathways associated with metastasis were AHI’s mechanism in BC treatment.

### Comprehensive Bioinformatics Analysis of GPX1

**FIGURE 4:**
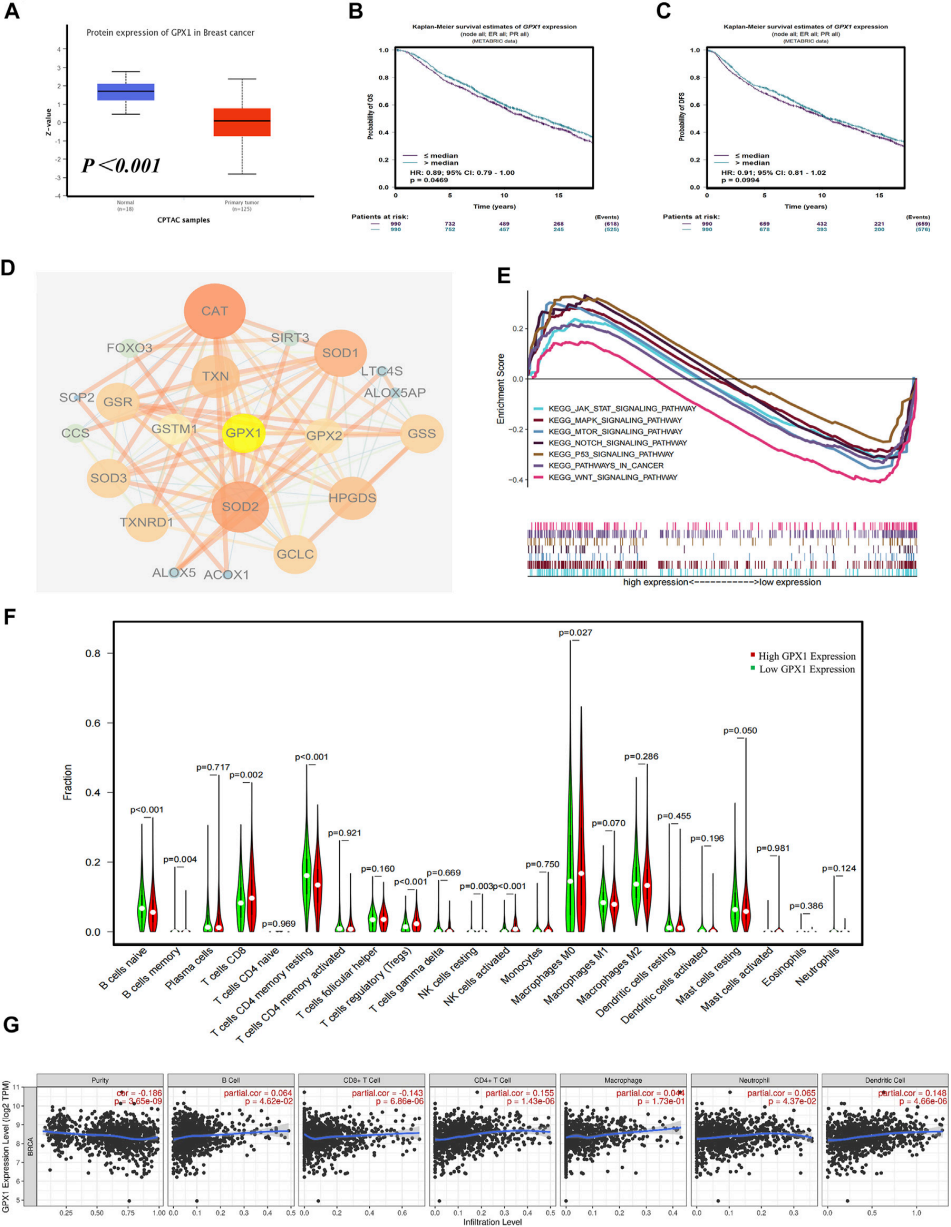
Comprehensive bioinformatics analysis of GPX1. **(A)** Analysis of the protein expression of GPX1 between normal breast tissue and BC tissue. **(B)** The OS and **(C)** DFS of patients in the high and low GPX1 expression groups. **(D)** The reciprocal proteins of GPX1. **(E)** Related pathways of GPX1 by GSEA. **(F)** The infiltration levels of immune cells in the high and low GPX1 expression groups in patients with BC. **(G)** Correlation between GPX1 and the abundance of immune infiltration.

We conducted a multidimensional analysis of GPX1 through bioinformatics analysis. Compared with normal breast tissue, the protein expression of GPX1 is lower in BC (*p* < 0.001) (Figure 4A). The overall survival (OS) of BC patients in the high GPX1 expression group was longer than those in the low GPX1 expression group (hazard ratio [HR] = 0.89, *95% CI* 0.79–1.00, *p* = 0.0469) (Figure 4B), indicating a prognostic value of GPX1. However, the high GPX1 expression did not exhibit considerable advantages in DFS (HR = 0.91, *95% CI* 0.81–1.02, *p* = 0.0994) (Figure 4C). The proteins that interacted with GPX1 mainly include SOD1, SOD2, SOD3, GCLC, TXN, and CAT (Figure 4D). The GSEA data showed that GPX1 may play roles in the JAK-STAT, MAPK, mTOR, NOTCH, P53, and WNT signaling pathways (Figure 4E). The above signaling pathways are similar to our previous transcriptome results.

The immune infiltration analysis also showed that tumors with high GPX1 expression levels had more infiltrated CD8+T, Tregs, M0 macrophages, and natural killer cells. By contrast, naive B cells and CD4+T cells were significantly reduced (*p* < 0.05) (Figure 4F). The expression of GPX1 in BC is related to the abundance of immune infiltration and the immune score (Figure 4G, Supplementary Figure S2).

### GPX1 is a Direct Target Protein of AHI in BC Cells

**FIGURE 5:**
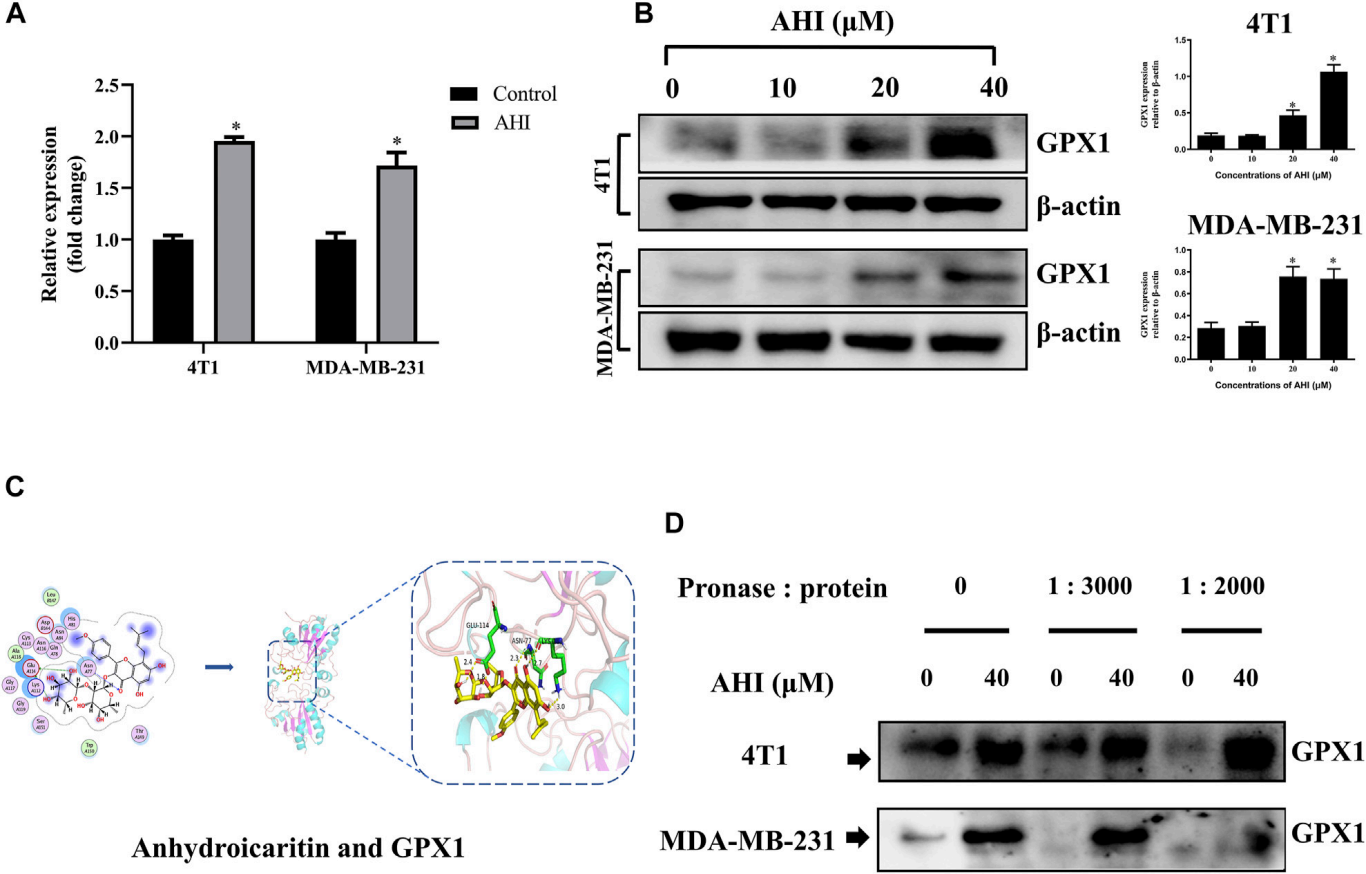
GPX1 was the drug target of AHI for treating BC cells. **(A)** Relative GPX1 expression analyzed by RT-PCR after AHI treatment in the 4T1 and MDA-MB-231 cells. **(B)** Protein expression of GPX1 after treatment with different concentrations of AHI (*p < 0.05). **(C)** Combined prediction model of AHI and GPX1 domain. **(D)** DARTS assay was performed to test the direct binding of AHI to GPX1 in BC cells. *p < 0.05.

To confirm the GPX1 identified by RNA-Seq, RT-PCR validation was performed with the 4T1 and MDA-MB-231 cell lines. After 24 h of AHI (40 μM) treatment, the GPX1 expression levels were significantly higher than those of the control (Figure 5A). Similarly, the protein expression of GPX1 gradually increased with the increased AHI concentration (Figure 5B). Molecular docking and DARTS analyses were conducted to further confirm whether the GPX1 was the solid drug target of AHI. The molecular docking results suggested a high affinity for docking between GPX1 and AHI (affinity = −7.62 kcal/mol) (Figure 5C, Supplementary Table S2). The DARTS experiment suggested that after AHI treatment, the GPX1 showed more stable property with pronase compared with that in the control group (Figure 5D). These results suggested that GPX1 may serve as a potential AHI target.

### GPX1 Is Negatively Correlated to the Malignant EMT in BC

**FIGURE 6:**
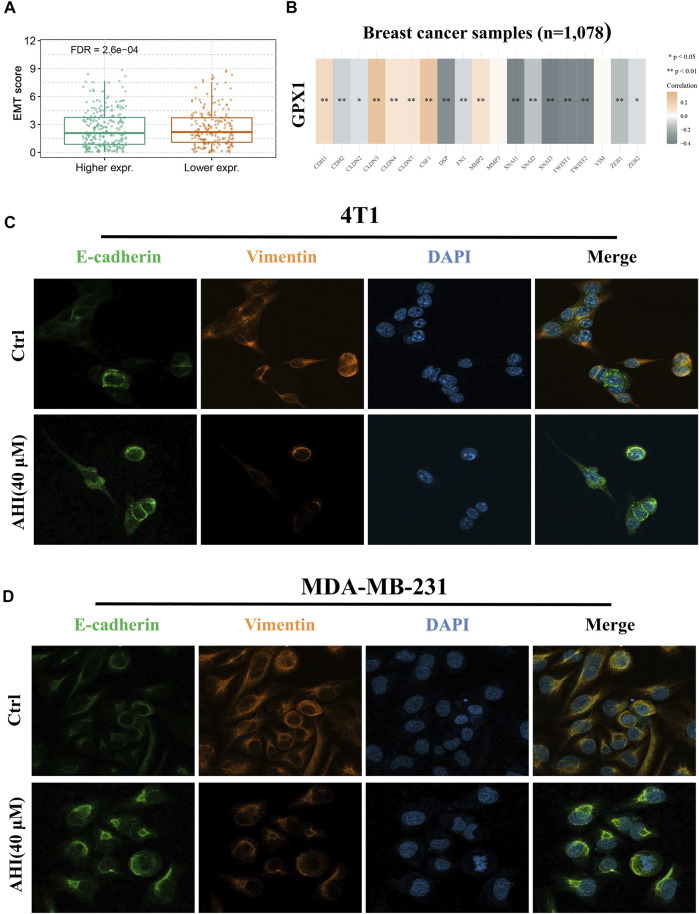
Identification of AHI-inhibited EMT by enhancing GPX1 expression. **(A**, **B)** Relationship between GPX1 expression and EMT. **(C**, **D)** AHI significantly increased E-cadherin (green fluorescence) and decreased vimentin (orange fluorescence) expression (×630) by confocal immunofluorescence analysis.

To further verify the relationship between EMT and hub gene, the GSVA method was used to evaluate the EMT pathway score variation. After the evaluation of the EMT scores of 1,078 patients with BC, we found significantly decreased EMT scores in the high GPX1 expression group compared with those in the low GPX1 expression group (Figure 6A). Subsequently, we first quantified GPX1’s EMT induction ability to comprehensively identify EMT-related genes. This evaluation suggested a significantly positive correlation between GPX1 expression and E-cadherin, CLDN3, CLDN4, CLND7, CSF1, MMP2, and MMP3. GPX1 also showed significantly negative correlation with N-cadherin, Snail family, TWIST1, TWIST2, ZEB1, and ZEB2. These findings implied the potential antitumor role of GPX1 possibly *via* suppression of the malignant EMT in BC (Figure 6B).

### AHI Inhibits EMT in BC Cells

Confocal immunofluorescence analysis indicated that AHI markedly increased E-cadherin expression and decreased vimentin expression (Figures 6C,D). E-cadherin (green fluorescence) and vimentin (orange fluorescence) are mainly located in the cytoplasm, and DAPI (blue fluorescence) is located in the nucleus. The E-cadherin protein expression level in the AHI group was significantly upregulated, whereas the vimentin expression level was significantly downregulated compared with those in the control group. This result indicated that AHI may significantly inhibit the occurrence and development of EMT.

### AHI Enhances GPX1 Expression and Inhibits EMT in the BC Xenograft Mouse Model

**FIGURE 7:**
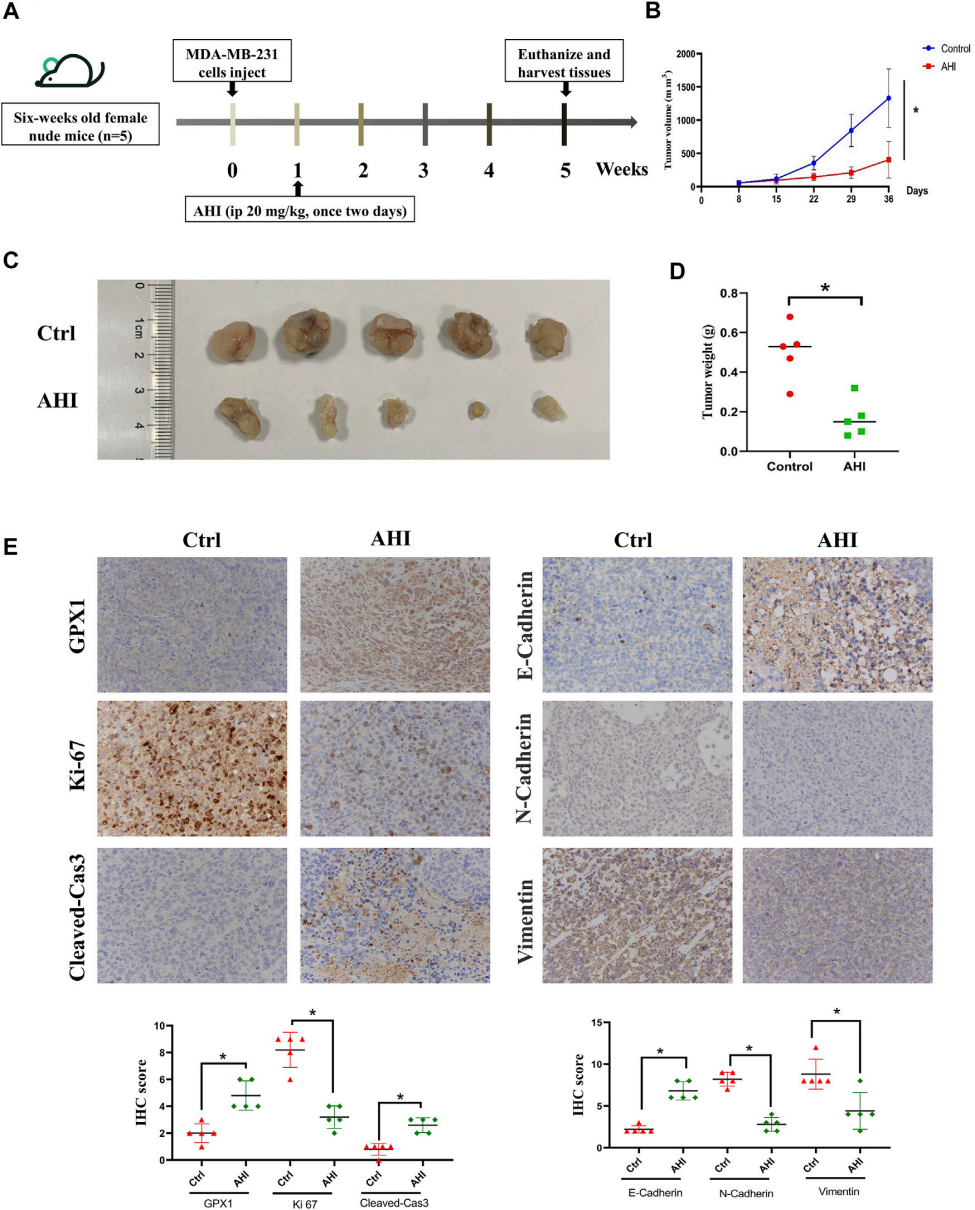
AHI enhances GPX1 expression and inhibits EMT in the BC xenograft mouse model. **(A)** Diagram showing the scheme for tumor implantation and AHI treatment. **(B**–**D)** BC proliferation after AHI treatment in the mouse model. **(E)** IHC analysis of GPX1, proliferation-related, and EMT-related proteins after AHI treatment in mice. *p < 0.05.

To assess AHI’s anti-BC effect, a xenograft model of the MDA-MB-231 cells was established in BALB/c nude mice (Figure 7A). After 28 days of treatment, the average tumor size of the control group was 1,332 ± 438 mm^3^ compared with 405 ± 275 mm^3^ of the AHI-treated group, which resulted in a significant decrease in tumor volume and weight (Figures 6B–D). AHI has no toxicity to the livers and kidneys of BC nude mice (Supplementary Figure S1). The IHC analysis revealed that AHI significantly downregulated the expression level of the proliferation-related proteins Ki-67 and upregulated the expression level of GPX1 and the proapoptotic proteins cleaved caspase3. In addition, the classical EMT-associated biomarker levels revealed that the expression level of the epithelial biomarker E-cadherin, which mediates cell-to-cell homogenous adhesion, significantly increased, whereas the expression levels of the interstitial markers, namely, N-cadherin and vimentin, which mediate cell-to-cell matrix heterogeneous adhesion, markedly decreased (Figure 6E).

## Discussion

BC has become one of the main risk factors leading to death in females (Ferlay et al., 2015). The incidence of BC gradually tends to be in the young population (Merino Bonilla et al., 2017). Endocrine therapy, targeted therapy, and chemotherapy are major treatments that significantly improve the prognosis of BC (Joseph et al., 2018; Zhao et al., 2020). However, BC is a complex heterogeneous disease with a large proportion of patients with progression, metastasis, and drug resistance (Echavarria et al., 2018).

This condition brings the current treatment into a bottleneck. We need to propose concepts of precise treatment to formulate an individualized BC treatment plan. The advances in genomics have made the establishment of individualized treatment options possible. Valuable methods to achieve targeted and individualized treatment plans for patients with BC include dimensional exploration of its biological nature, development of new targets based on omics data, and molecular biological characteristics.

GPX is a peroxidase containing selenocysteine. GPX consists of eight antioxidant enzymes, and their main function is to defend the organism against oxidative stress. A previous study revealed that GPXs have antitumor effects because they block reactive oxygen species (ROS) and regulate the redox signaling system, which plays a key role in tumor growth (Wu et al., 2021). Hydroperoxides stimulate cell proliferation and migration, but excessive hydroperoxides lead to cell apoptosis. The redox status of tumor cells therefore has a dual role. Downregulation of GPX1 expression levels leads to a decrease in the ability to respond to ROS, which is conducive to the accumulation of oxidative damage and promotes tumor progression (Méplan et al., 2013). GPX1 expression levels were found to be significantly downregulated by 7.4% in the tumorous breast tissue when compared with the nonmalignant one (Król et al., 2018). Lutein significantly inhibits the viability of BC cells, and the inhibition roles may be related to the upregulation of GPX1 expression and the downregulation of oxidative stress, thus blocking the nuclear factor κB signaling pathway (Chang et al., 2018).

As an antioxidant gene, the inhibitory effect of GPX1 on EMT has also been reported previously. GPX1 inhibits the EMT by regulating the Akt/GSK3β/Snail signaling axis in pancreatic adenocarcinoma (PDAC). Moreover, low GPX1 expression levels correlated with a poor survival rate in patients with PDAC (Meng et al., 2018). SEPP1 may inhibit the proliferation of HCC cells, accompanied by a decrease in ROS production and an increase in GPX1 expression (Wang J. et al., 2020). Glutathione metabolism and glutathione itself could be positive targets to prevent EMT in non–small cell lung carcinoma (Matadamas-Guzman et al., 2020). Changes in GPX1 expression may be related to EMT in CC cells (Yoo et al., 2009). Research on this matter is limited, considering that GPX1 serves as an oncogene in cancers (Gong et al., 2021; Lee et al., 2021). However, few studies have been conducted on GPX1 expression and EMT in BC.

*E. brevicornum* is a Chinese herbal medicine whose main functions are to nourish kidney yang, strengthen muscles and bones and dispel rheumatism. The main active ingredient of *E. brevicornum* is icariin. AHI is the main metabolite after icariin is taken orally into the body and decomposed by intestinal bacteria (Liu et al., 2008). AHI has antiosteoporosis (Zhai et al., 2011), antitumor (Nguyen et al., 2017), and antioxidant properties (Bao et al., 2012). AHI also significantly inhibits the activation of mouse macrophages and is a potential immunosuppressant (Lai et al., 2012).

Here, we found that the inhibition rates of 4T1 and MDA-MB-231 cells gradually decreased with varying concentrations of AHI. This finding indicates that AHI inhibits the survival of BC cells. By comparing the survival rates of 4T1 and MDA-MB-231 cells treated by different concentrations of AHI, 40 μM was identified to be the optimal concentration. 4T1 and MDA-MB-231 cells treated with this concentration were extracted for RNA sequencing. The result showed that AHI significantly enhanced GPX1 expression. The role of GPX1 expression in BC and its relationship with EMT remain unclear.

We thus performed a comprehensive biological analysis of GPX1 expression. We found that GPX1 expression relatively decreased in BC, and GPX1 expression was positively correlated with the survival duration. Analysis of GPX1 upstream and downstream regulatory proteins revealed that SOD1, SOD2, SOD3 (Yi et al., 2017; Li et al., 2018), GCLC (Matadamas-Guzman et al., 2020), and TXN (Cao et al., 2020) are related to EMT and cell migration and invasion. Network pharmacology analysis revealed that AHI’s main targets are SRC (Qin et al., 2021), NFKB1 (Xu et al., 2021), and EGFR (Jin et al., 2021), which have close relationships with EMT. The gene set variation analysis results showed significantly decreased EMT scores in the high GPX1 expression group compared with those in the low GPX1 expression group. The significant correlation between GPX1 and EMT-related genes was detected through comprehensive identification.

We then confirmed the promotion of AHI on GPX1 from protein and gene expression *via* Western blot analysis and RT-PCR experiment. Molecular docking and DARTS experiments also proved that AHI and GPX1 bind well. The EMT pathway score variation results show that GPX1 expression possibly suppresses the malignant EMT in BC. Through the above research, we proposed a preliminary conjecture that AHI can inhibit EMT by enhancing GPX1 expression and play an anti-BC effect. Further animal experiments showed that the final tumor volume and weight of the AHI group were smaller compared with those in the control group. The IHC analysis showed that AHI could reduce the expression of Ki-67, N-cadherin, and vimentin, while increasing the expression of GPX1, E-cadherin, and cleaved caspase3.

Immunotherapy has become an emerging therapy in cancer treatment. Programmed cell death protein-1 and programmed cell death protein 1 ligand inhibitors can benefit patients with metastatic BC (Page et al., 2019). However, the immunotherapy of BC remains a major challenge to medical research (Jiang et al., 2021; Tabana et al., 2021). In the present study, we found that GPX1 is related to the immune infiltration of BC. Whether GPX1 can be used as a BC-related immune checkpoint remains to be further verified.

## Conclusion

**FIGURE 8:**
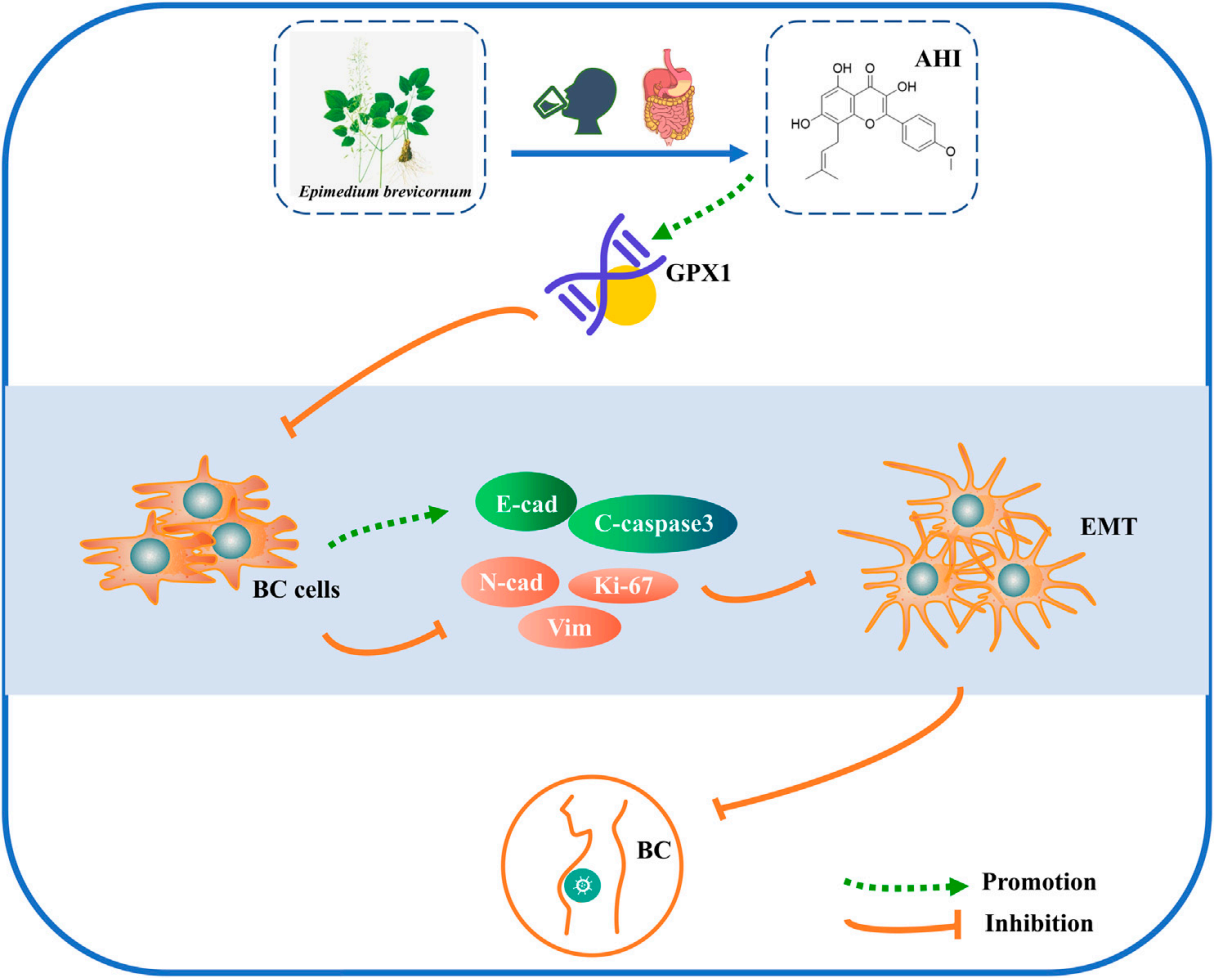
Schematic diagram of the mechanism possibly involved in AHI-mediated BC.

The present study determined a potential mechanism of AHI against BC by conducting bioinformatics and experimental studies. AHI plays an anti-BC effect by inhibiting EMT progression. The potential mechanism may be related with enhancing the expression of GPX1, E-cadherin, and cleaved caspase3 and inhibiting the expression of N-cadherin, vimentin, and Ki-67. AHI may significantly upregulate GPX1 expression in BC, thus effectively suppressing EMT (Figure 8).

## Data Availability

The datasets presented in this study can be found in online repositories. The names of the repository/repositories and accession number(s) can be found below: NCBI (accession: SAMN21399181-SAMN21399192).
