# Supplementary material for: Anhydroicaritin Inhibits EMT in Breast Cancer by Enhancing GPX1 Expression: A Research Based on Sequencing Technologies and Bioinformatics Analysis
Source: Front Cell Dev Biol. 2022 Feb 1;9:764481. doi: 10.3389/fcell.2021.764481 (PMC8844201; doi:10.3389/fcell.2021.764481)
Supplement: Supplementary file 1 [file DataSheet1.doc]

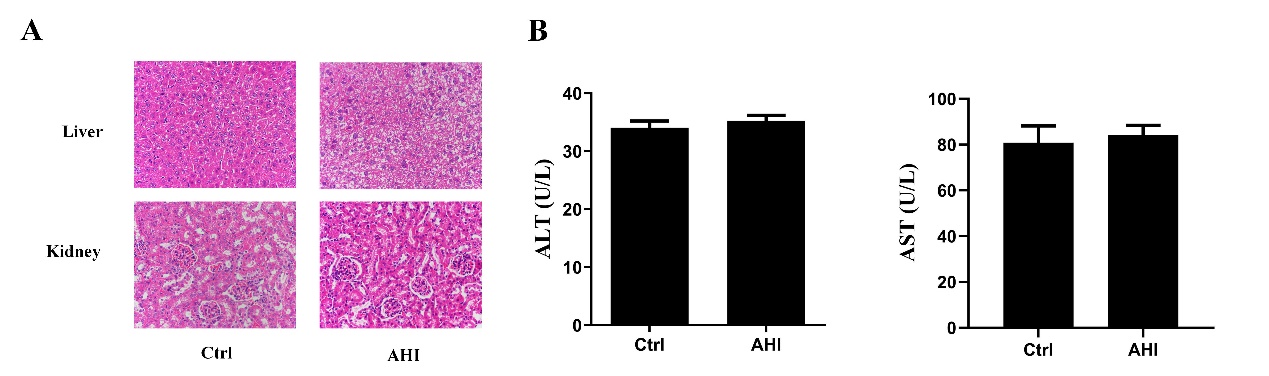


**Supplementary Figure 1 |** AHI has no toxicity to liver and kidney in breast cancer nude mice. (A) HE staining showed that there was no difference in the structure of liver and kidney between AHI and control group. (B) There was no statistical difference in ALT and AST between the AHI and the control group.
