# Supplementary material for: Anhydroicaritin Inhibits EMT in Breast Cancer by Enhancing GPX1 Expression: A Research Based on Sequencing Technologies and Bioinformatics Analysis
Source: Front Cell Dev Biol. 2022 Feb 1;9:764481. doi: 10.3389/fcell.2021.764481 (PMC8844201; doi:10.3389/fcell.2021.764481)
Supplement: Supplementary file 2 [file Table1.doc]

| Name | Primer | Sequence information |
| --- | --- | --- |
| GPX1-human | Forward Primer | CAGTCGGTGTATGCCTTCTCG |
|  | Reverse Primer | GAGGGACGCCACATTCTCG |
| GAPDH-human | Forward Primer | AAAATCAAGTGGGGCGATGC |
|  | Reverse Primer | TGGTTCACACCCATGACGAA |
| GPX1-mouse | Forward Primer | CCCGTGCAATCAGTTCGGA |
|  | Reverse Primer | TAAAGAGCGGGTGAGCCTTC |
| GAPDH-mouse | Forward Primer | AGGTCGGTGTGAACGGATTTG |
|  | Reverse Primer | TGTAGACCATGTAGTTGAGGTCA |

**Supplementary Table 1|** Specific information of primers of RT-PCR.
