# Supplementary figures and images for: Anhydroicaritin Inhibits EMT in Breast Cancer by Enhancing GPX1 Expression: A Research Based on Sequencing Technologies and Bioinformatics Analysis
Source: Front Cell Dev Biol. 2022 Feb 1;9:764481. doi: 10.3389/fcell.2021.764481 (PMC8844201; doi:10.3389/fcell.2021.764481)

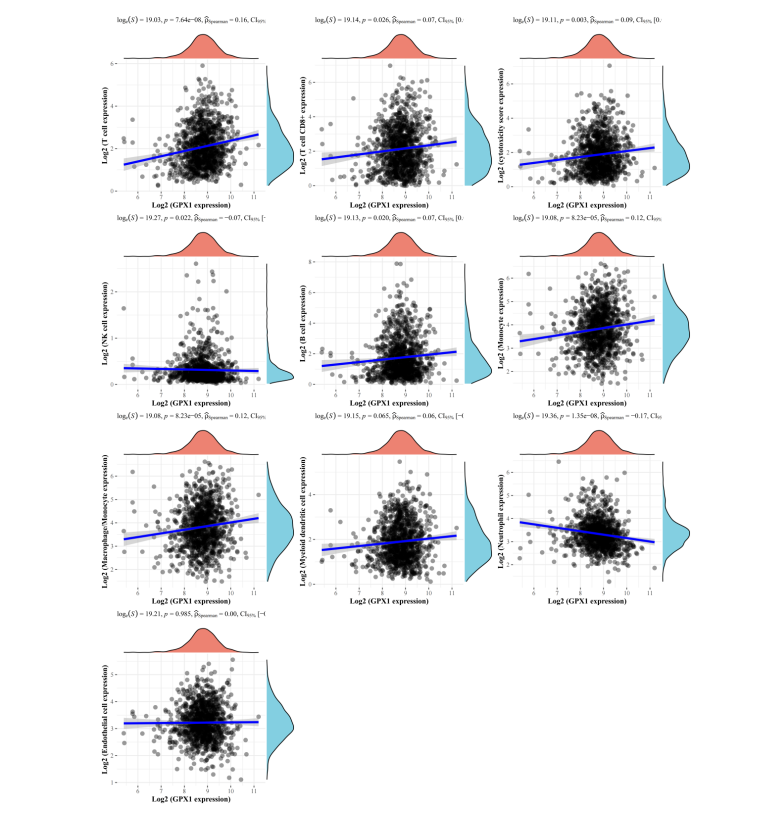


**Supplementary Figure 2 |** Spearman correlation analysis between GPX1 and immune score.

Supplement: Supplementary file 3 [file DataSheet2.doc]
