# Supplementary material for: Anhydroicaritin Inhibits EMT in Breast Cancer by Enhancing GPX1 Expression: A Research Based on Sequencing Technologies and Bioinformatics Analysis
Source: Front Cell Dev Biol. 2022 Feb 1;9:764481. doi: 10.3389/fcell.2021.764481 (PMC8844201; doi:10.3389/fcell.2021.764481)
Supplement: Supplementary file 4 [file Table2.doc]

| Name | Compound  structure | Binding energy  (kcal/mol) | Combination  type |
| --- | --- | --- | --- |
| Anhydroicaritin |  | -7.62 | Hydrogen bonds,  Hydrophobic interactive |

**Supplementary Table 2 |** GPX1 docking results for Anhydroicaritin.
